# The timing and spatial distribution of mother–offspring interactions in an obligate hider

**DOI:** 10.1186/s40462-024-00514-5

**Published:** 2024-11-26

**Authors:** Sophie Baur, Ferdinand P. Stehr, A. J. Mark Hewison, Nicolas Morellet, Nathan Ranc, Andreas König, Annette Menzel, Wibke Peters

**Affiliations:** 1 Research Unit Wildlife Biology and Management, Bavarian State Institute of Forestry, Hans-Carl-von-Carlowitz-Platz 1, 85354 Freising, Germany; 2 Professorship of Ecoclimatology, TUM School of Life Sciences, Technical University of Munich, Hans-Carl-von-Carlowitz-Platz 2, 85354 Freising, Germany; 3 Wildlife Biology and Management Unit, TUM School of Life Sciences, Technical University of Munich, Hans-Carl-von-Carlowitz-Platz 2, 85354 Freising, Germany; 4 INRAE, CEFS, Université de Toulouse, 31326 Castanet-Tolosan, France; 5 LTSER ZA PYRénées GARonne, 31320 Auzeville Tolosan, France; 6 Institute for Advanced Study, Technical University of Munich, Lichtenbergstraße 2a, 85748 Garching, Germany

**Keywords:** *Capreolus capreolus* L., Maternal allocation, Roe deer, Rearing strategy, Nursing behavior

## Abstract

**Background:**

Parental care is indispensable for the survival and development of dependent offspring, often requiring a delicate balance of time and energy allocation towards offspring by parents. Among ungulates employing a hider strategy, deciding when and where to provide care while also maintaining a sufficient distance to not reveal the offspring´s hiding place is likely crucial in determining their fate.

**Methods:**

In this study, we analyzed the timing and spatial distribution of mother–offspring interactions in roe deer females (*Capreolus capreolus L.*). We fitted roe deer mothers and their neonates with GPS-collars combined with a proximity sensor in south Germany to address the spatial and temporal distribution of mother-fawn interactions during the first two months of the fawns’ lives.

**Results:**

We observed variations in the distance between mother and fawn, which initially increased over the first month and then decreased as the fawns grew older. The timing of mother-fawn contacts was strongly linked with the circadian rhythm of the mother, aligning closely with their typical bimodal activity peaks at dawn and dusk. Furthermore, we observed differences in habitat use between mother and offspring, reflecting the mother's requirements for food and protection (e.g. greater use of forests, higher distances to roads), as well as the fawn's priority requirement for protection (e.g. higher use of unmown grassland). We documented variations over time, highlighting how these requirements changed as the fawn ages. Interestingly, during the initial two weeks, most of the contacts occurred in habitats that were particularly favored by mothers. However, as the fawns aged, contacts occurred increasingly often in habitats that were routinely used by fawns.

**Conclusions:**

Understanding the timing, frequency, and spatial distribution of mother–offspring interactions provides valuable insights into the care strategies of hider ungulates. The observation that mothers leave their fawns in agricultural fields during the first few weeks of life has strong implications for wildlife management, as this behavior constitutes a kind of evolutionary trap under current agricultural practices and mowing regimes. Whether females can adjust their maternal care tactics to these novel selection pressures in human-altered landscapes is likely key to predicting the population dynamics of this obligate hider.

**Supplementary Information:**

The online version contains supplementary material available at 10.1186/s40462-024-00514-5.

## Background

In mammals, parents, and commonly females, must allocate significant energy and time caring for their offspring [21]. Parental care is a key determinant of fitness as it enhances the likelihood of offspring survival and, in turn, the chances of successful reproduction of the offspring [21, 80]. However, care is costly, and parents face their own physiological demands for maintenance. Especially under limiting conditions, mammals generally adopt a conservative strategy that prioritizes their own survival over their neonates [30, 33, 84]. Therefore, parents must carefully balance energy allocation between caring for their offspring and ensuring self-maintenance to maximize their current and future reproductive success [3].

In ungulates, rearing strategies have been classified into two main categories: the follower and the hider strategies [54]. In hider species offspring remain hidden in a location with sufficient protection most of the time, with frequent but short mother–offspring interactions for bouts of intensive maternal care [54]. The timing and duration of these interactions, as well as the distance between mother and offspring when hiding influence rearing success. In this situation, the mother’s choice of where to care for her offspring within her home range is critical to satisfy the female´s high nutritional requirements to support lactation and maintenance [69], along with the requirements of the vulnerable offspring for rapid growth and safe hiding places [4].(Especially for income breeders, like roe deer (*Capreolus capreolus*), that do not maintain substantial energy reserves [5], females must obtain the required energy to finance reproduction directly from current acquisition of high-quality resources [45].

Due to their high behavioral plasticity and dietary flexibility, roe deer can fulfill their nutritional and safety requirements in diverse habitats, including large forests, open habitats, and mixed landscapes (e.g. [4, 24, 40, 64, 85]). However, in addition, fawns need sufficient cover to hide from possible predators, especially during the first weeks of life (e.g. [72]) and vegetation structure that provides a suitable microclimate for thermoregulation [51]. Besides the natural predation risk for neonates, particularly from fox (*Vulpes vulpes*) [1, 57], human land-use practices such as spring mowing are a major source of mortality for fawns in mixed agricultural landscapes [42]. This risk is particularly high during the first 2–3 weeks of life, when the hiding reflex is very pronounced and flushing probability low [50, 59].

Within the range of the rearing habitat that is selected by the mother, the neonate chooses where to hide, independently from the mother, on a very small spatial scale [7]. However, this choice is partially dependent on the mother, as it is constrained by her spatial behavior on a larger spatial scale [54]. In an obligate hider, habitats where mother and offspring meet for parental care, e.g., for nursing, may be mutually preferred (i.e., they are selected habitats for both mother and offspring) or, alternatively, one or the other must compromise their primary habitat choices and, hence, potentially pay some costs. Moreover, finding a suitable balance in terms of where and when mother and offspring interact is key for a successful hiding strategy. In the case of roe deer, fox predation on neonates can be high [1, 57]. Hence, close proximity between mother and fawn can be beneficial for potential defense against predators [43, 44, 56], while on the other hand, maintaining an appropriate distance to avoid revealing the location of the offspring to predators may be equally important [19].

To our knowledge, research is scarce in the context of the rearing tactics of a hider species regarding the spatial location and timing of contacts between mother and offspring, in relation to habitat heterogeneity. Previous studies have often focused on contrasting care behaviors with those of non-reproductive individuals, or comparing pre- and post-parturition behavior of females and neonates separately (e.g., [9, 16, 29, 60, 87]). One contributing factor to this knowledge gap may be the limited technological capacities, which have historically hindered the gathering of comprehensive and precise data simultaneously from both offspring and mother, as well as their interactions. Previously, to address these questions, studies required extremely high intensity of fieldwork and often relied on direct observations, but often provided an incomplete picture [48, 88]. However, advancements in GPS satellite telemetry and proximity logger technology have made the collection of comprehensive data feasible, even for small ungulates like roe deer.

Here, for the first time to our knowledge, we used advanced GPS- and proximity technology with a very high spatiotemporal resolution to analyze the timing and spatial distribution of interactions between roe deer mothers and their neonates in an obligate hider. Specifically, we described how the distance between mother and offspring varied over the period of intensive care, as the fawn aged. We expected that mothers would maintain a lower distance conducive to defending the fawn during the strict hider phase (first 2–3 weeks) [19, 44, 50, 56], when they are most vulnerable to predation. In contrast, we expected this initial distance between mothers and offspringto increase with fawn age, but then decrease as the fawn becomes more active and able to follow its mother [59]. We further predicted that encounters between mothers and fawns i.e., for nursing and caregiving, would be unevenly distributed over the day [13]. In hider species, the mother generally initiates contacts [29, 35]. Hence, we expected mother-fawn interactions to be driven by the habitual circadian rhythm of the females, with bimodal activity peaks at dawn and dusk [9]. Next, we contrasted the space use of roe deer mothers with that of their fawns to describe the habitat types in which their encounters occurred. Because we expected differences in habitat use reflecting their respective needs, we expected maternal habitat use to be mainly determined by their nutritional demands during lactation [69], with a higher tolerance of risk (to human disturbance) as long as potential refuges were available nearby [7, 85]. In contrast, we expected fawns to mainly seek protective cover, provided by high-ground vegetation and a low level of potential human disturbance [7, 58, 60]. Lastly, we investigated which habitats were predominantly used for mother–offspring interactions and whether these locations corresponded more closely to the preferred habitats of mothers or fawns. As ungulate females adopt a conservative maternal care strategy, implying that they favor their own survival and maintenance over current allocation to reproduction [33], we expected roe deer mothers to give birth in locations that mainly meet their needs [30, 84], but as the fawns grow older and become more independent, we expected more variation in the spatial distribution of mother-fawn interactions.

## Materials and methods

### Study areas

**Fig. 1:**
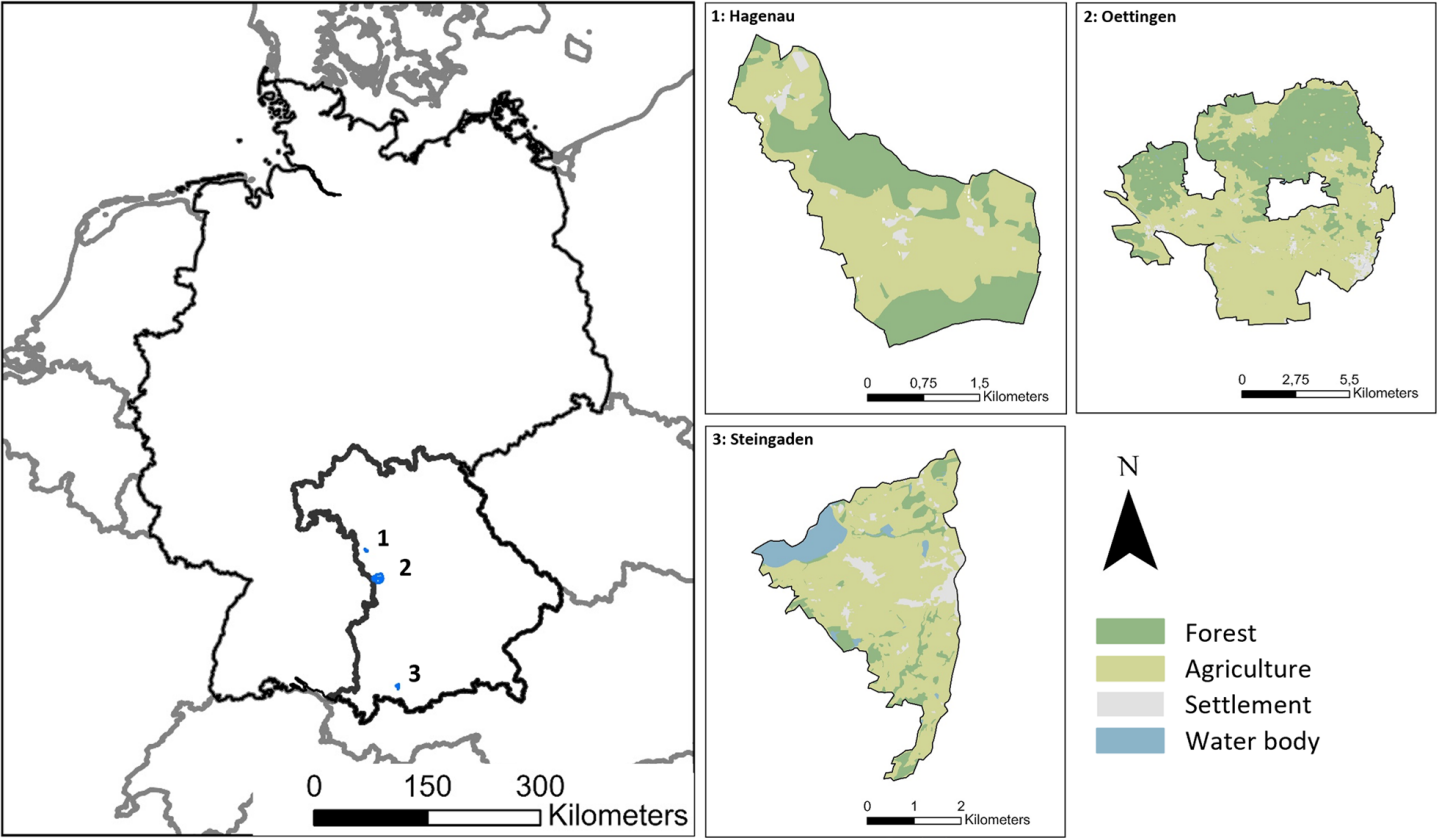
Location of the study areas in central-western and southern Bavaria, Germany (left), and the respective main land use types for each area (right)

We collected movement and proximity data for female roe deer fawn pairs in three study areas located in the central-western (Oettingen: N 48.98, E 10.53 / 410–450 m a.s.l; Hagenau: N 49.31, E 10.32 / 430–450 m a.s.l) and southern (Steingaden: N 47.69, E 10.84 / 750–800 m a.s.l.) parts of the federal state of Bavaria, Germany. In the two central-western areas, agriculture was dominated by crops, bio-energy production, and grassland (Oettingen: 37% forest, 51% agricultural area; Hagenau: 37% forest, 59% agricultural area [2]). In the south, Steingaden was comprised of 13% forest and 76% agriculture [2], mainly permanent grassland used for grass, silage, or hay production (Fig. 1).

### Data collection and processing

Between 2021 and 2023 we captured 49 adult roe deer females (age > = 2 years) using net traps [27] and box traps [10] during winter (November-March). Females were equipped with Lotek GPS-collars (LiteTrack 360, LOTEK WIRELESS INC, Newmarket, Canada). Collars were scheduled to obtain one location every 15 min between April 15th and the end of June and one location every 2 h during the remainder of the year. For four females, data for more than one annual reproductive event were available. To identify the date of birth, we monitored the movement patterns of radio-collared females and observed them repeatedly during the birth period, from the end of April to June. To locate their fawns in the field, we observed females from a distance or searched for individual fawns with thermographic cameras attached to unmanned aerial vehicles [68]. Especially in forested areas, we used a systematic search around the GPS cluster site. Once a fawn was detected, we captured it by hand and age was estimated in accordance to Stehr et al. (under review) based on umbilical cord, body weight, hind foot, and body length.

We radio-collared fawns that were estimated to be older than 24 h and heavier than 2 kg with Litetrack PinPoint tags (PinPoint 450, LOTEK WIRELESS INC, Newmarket, Canada) attached to a small expandable collar (Followit, Lindesberg, Sweden) (n = 27). Fawn GPS tags were generally scheduled to collect one fix per hour, with bursts for some periods, every 30 min (during mowing periods) or six hours (when the fawn became older). Capture and handling of females and fawns were approved by the government of Lower Franconia, Germany, in accordance with German law and animal welfare regulations (capture permit RUF-55.2.2-25322-1160-25). We conducted genetic analyses retrospectively to validate that mothers and fawns were paired correctly. DNA was extracted from hair samples manually using the QiaAmp DNA micro kit (Qiagen, Hilden, Germany) and from tissue samples using the Chemagic DNA tissue kit and a MSM1 Magnetic Separator (Perkin Elmer, Rodgau, Germany). For detailed genetic methods and marker set see Ebert et al. [28]. All genetic analyses were carried out by the wildlife genetic laboratory Seq-IT GmbH & Co. KG (Kaiserslautern, Germany).

Mortality of fawns and radio-collar failure reduced our sample size progressively over the summer. For subsequent statistical analyses, we used pairs of mothers and fawns with a minimum length of 10 days of simultaneous monitoring. Our GPS dataset, thus, comprised 21 pairs (2021: n=10, 2022: n=8, 2023: n=3) hereafter referred to as *GPS-data*. We focused our analyses on the first 60 days post-partum for the reasons mentioned above and to limit overlap with the mating season that generally occurs during late July and early August [25]. The duration of the GPS data per female-fawn pair and the age of the fawn at the time of collaring are shown in Additional file: Table A1. We applied a DOP (Dilution of Precision) filter of ≤ 5 to reduce location error by excluding major outliers [55].

Fourteen female and fawn collars/tags were additionally carried integrated with proximity sensors, which recorded a proximity event when both collars encountered each other in the field. The proximity settings allow the recording of two distinct datasets. First, in the *GPS-proximity-data*, each proximity record consisted of the same information as the regular female collar records (xy-coordinates, date, time, DOP, etc.). Second, the *proximity-contact-dataset* recorded the start and end time of a proximity event, the duration, the ID of the contacted fawn tag, and the average RSSI (relative signal strength indicator; dBm). We set the RSSI to a threshold of -80 dBm and the burst rate to 20 bpm, ensuring that only contacts with a signal strength greater than that were recorded and transmitted, representing contacts between doe and fawn of approximately 2 m or less. Further, we set the separation time of recorded contacts to 10 min, hence successive contacts were only recorded as a new contact after 10 min elapsed in both data sets.

### Environmental descriptors

To describe land use, we obtained agricultural land use types from the Invekos Database between 2021 and 2023 [8]. Other land use types were derived from the Atkis land use system [2] and both merged into one layer (categories: grassland, maize, uncultivated, forest, other, winter crop (set as reference category)). Since the availability of vertical and horizontal cover provided by the ground layer changed significantly during the study period, especially for fawns, due to grassland mowing practices and phenology, we linked all grassland areas with the corresponding mowing events detected using the approach of Reinermann et al. [76]. We classified grassland as "mown" (minimal cover) following a mowing event for up to 3 weeks and the remaining weeks until the subsequent mowing event as "unmown".

We further used the Atkis database to extract forest and hedge cover and manually post-digitized missing hedges. Both layers were merged, and the distance to the nearest wooded patch in m was calculated in ArcGIS Pro 2.9 (ESRI, Redlands, CA, USA). Because roe deer are considered to preferentially use ecotone habitats at the forest-field boundary [85], we generated a metric where negative distances correspond to locations within the forest patch up to the forest edge, the edge itself was assigned a value of 0, and distances outside the forest patch were represented by increasingly positive values. The raster layer characterizing the canopy roughness of wooded structures (1 × 1 m) was generated using the methodology outlined by Kirchhöfer et al. [46]. This layer depicts the standard deviation of height values of the ground layer per pixel, where each pixel represents 20 × 20 m. Using the "osmdata" R package [71], we extracted all paved and unpaved roads within the study areas, and computed the distances for each raster cell to the nearest road in ArcGIS. We processed all raster layers to a resolution of 5 × 5 m and, for ease of comparing effect sizes, all continuous variables were z-transformed.

### Spatio-temporal separation of mothers and their fawns

#### Distance between mother and fawn

**Fig. 2:**
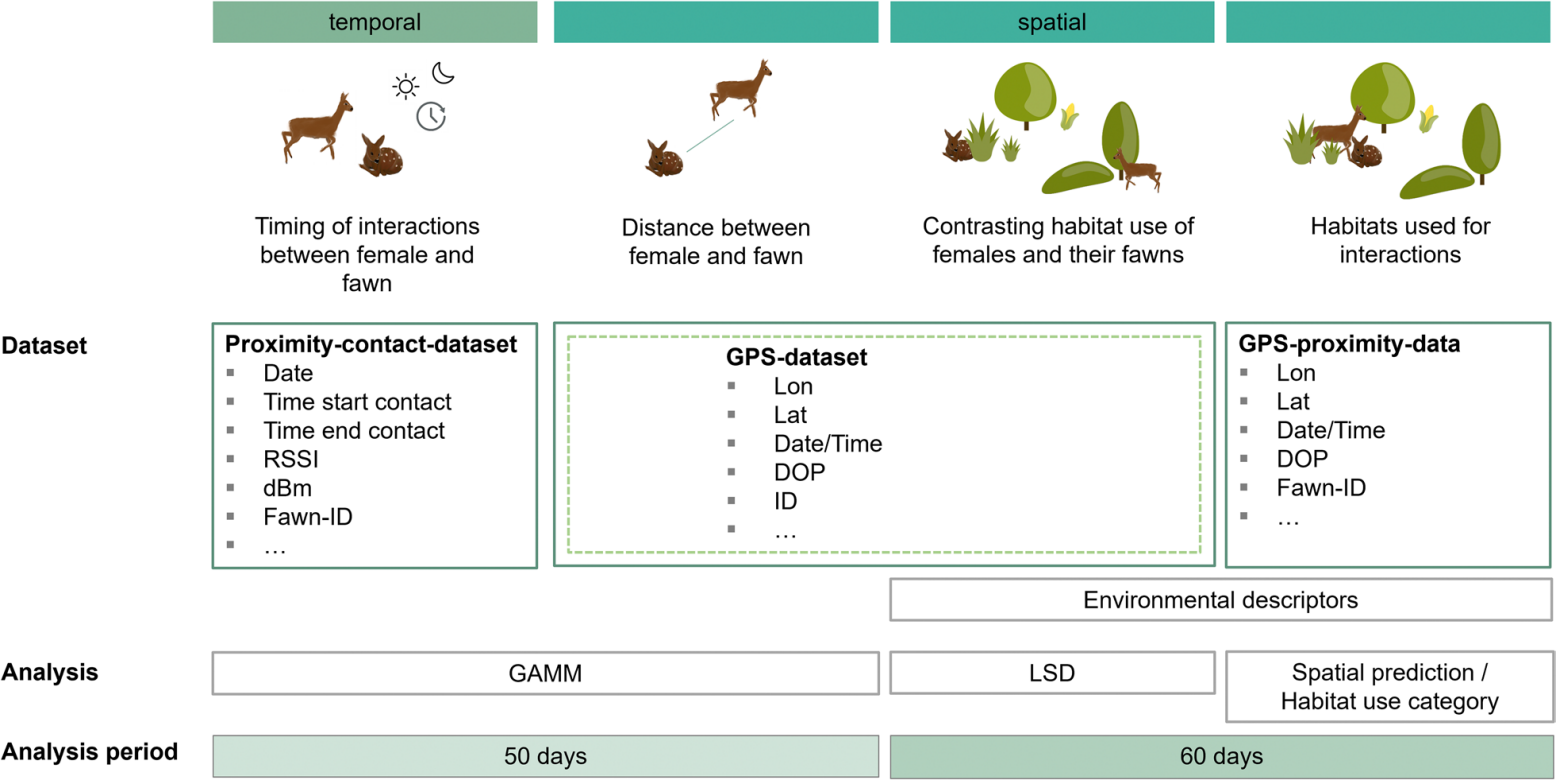
Overview of the dataset, statistical analysis, and the period analyzed to study mother–offspring interactions in roe deer. The dark green boxes describe the data recorded by the female collar, while the light dashed line indicates data recorded by the fawn tag

We compared location data between each mother–offspring pair to explore variations in the distance between mother and her fawn over time. Due to differences in the temporal resolution of the *GPS-data* within and between mother-fawn pairs, all trajectories were first re-sampled to a 1-h interval using the "adehabitat" package [20]. Next, the distance between the GPS location data of each female and her corresponding fawn were calculated at the same respective times. We fitted a generalized additive mixed model (GAMM, [38]) with the "mgcv" package [89] to describe variation in the mean distance (log-transformed) between mother and fawn, including a thin plate regression spline on the fawn´s age (in days). We included a random intercept for each mother–offspring pair to account for repeated observations. Due to the sensitivity of the models to data loss resulting from collar failure or mortality, only mother–offspring pairs with a monitoring duration until a fawn age of 50 days were included in this analysis (Fig. 2). This yielded 13 mother-fawn trajectories out of the initial 21 pairs. All statistical analyses were conducted in RStudio (Version 4.2.3).

#### Timing of interactions between mother and fawn

To analyze when interactions between a mother and her fawn occurred over the day, we equally restricted the *proximity-contact-data* to a maximum monitoring period of 50 days, and with a minimum of at least 10 days. The model did not show the same sensitivity to early mortality, allowing us to include all mother–offspring pairs with proximity data (14 pairs). Further, we counted the number of contacts per 15 min during the analysis period and modeled this metric in relation to the 24-h day cycle using a GAMM. Here, we modeled proximity as the number of contacts with a cyclic spline (on time of the day), with pair-ID included as a random effect on the intercept. We also explored the variation in mother–offspring proximity expressed as an aggregation of contacts per hour, with a zero-inflated model to account for the periods without any contacts. All statistical models yielded comparable results.

### Contrasting relative habitat use of females and their fawns

To contrast mother and fawn pairs (i.e. relative use), we tested for differences in frequency of land use types, distance to wooded patches, canopy roughness, and distance to roads (paved and unpaved) at used locations by mother and fawn (Sect. "Environmental descriptors"). We fitted logistic regression models to estimate coefficients for latent selection difference functions (LSD, R-package: glmmTMB, Version: 1.1.8; [15]). In an LSD, the model coefficients can be interpreted as the difference in habitat use between two groups relative to each other. This does not correspond to a classical analysis of habitat selection sensu stricto in that we did not analyze habitat use in relation to availability. Instead, because “availability” for one group of animals (i.e. fawns) is actually the habitat use of a second group (i.e. mothers), we herein refer to this as “*relative* habitat use”. LSD functions are frequently used to contrast resource use between individuals of two species, seasons, age-classes or sexes (e.g., [32, 66, 83]). We coded mothers as 1 and fawns as 0, hence, an increase in the parameter estimate for a given environmental descriptor indicates that the relative use by mothers was higher compared to that of their fawns.

As we expected changes in relative habitat use as the fawns grew older, we analyzed the nonregularized data (*GPS-data*) in four age classes (m = model), each constituting a 14-day interval (i.e., m1 = age 1–14, m2 = age 15–30, m3 = age 31–45, m4 = age 46–60). To avoid biasing parameter estimates towards mother–offspring pairs or individuals with a larger number of localizations, we included a weighting factor per individual based on sampling duration and intensity, as outlined in Mueller et al. [66]. Depending on the monitoring duration, we analyzed between 16 and 21 mother–offspring pairs per age class. Prior to the analysis, we tested for correlation among explanatory variables and, when correlated (Spearman´s Rank correlation |*r*|≥ 0.60), we selected the variable with the strongest explanatory power based on model comparison with one or the other variable using AIC (Akaike´s Information Criterion, [18]). Following this approach, we excluded canopy roughness from all subsequent analyses (Additional file: Table A2). To account for potential nonlinear relationships with continuous variables, we additionally tested for quadratic versus linear terms using GLMMs by assessing their fit and selected them based on the smallest AIC for univariate models.

Lastly, to interpret the magnitude of the differences in relative habitat use between mothers and their fawns, we calculated the relative selection strength (RSS) associated with LSD coefficients by estimating the relative use of one location versus a second location while holding the other predictors constant [6, 31]. To this end, we used the log_rss function in the "amt" package [82]. Usually, positive log-RSS values indicate selection, negative values indicate avoidance, and values equal to 0 neither preference nor avoidance [6, 31]. In our case, using LSD to contrast habitat use of mother and fawn, we interpreted positive values as indicating higher relative use by mothers compared to their fawns, and respectively, negative values indicated lower relative use by mothers compared to their fawns.

#### Habitats used for interactions

To evaluate the types of habitats where mother–offspring interactions occurred preferentially, i.e., those habitats used relatively more by mothers versus those used relatively more by fawns, we used the computed LSDs (describing the spatial separation between mother and fawn) to generate spatial predictions, which were then visualized as geographic maps, as outlined in Peters et al. [73]. These spatial prediction maps reflect the relative probability that a raster location was used relatively more or less by a female versus her fawn. Given that the types of vegetation grown in the agricultural areas differed between years and over time, we generated these spatial maps for each year and age class separately. Further, we calculated 14-day home ranges for each mother–offspring pair using minimum convex polygons (MCP, 95%, R-package "adeHabitatHR" [20]) equivalent to our age classes in the LSDs. Within these mother–offspring pair home ranges, we extracted the values of the corresponding spatial prediction map. We classified the extracted values into five equal-area quantiles to ensure comparability between years and age classes (habitat use category) [65]. Next, we extracted the categorical habitat use category for all recorded locations in the *proximity-contact-data*. This procedure should reveal whether they interact more often in areas used relatively more by mothers (value closer to 5) or by fawns (value closer to 1). Lastly, we compared the relative proportions of habitat use categories at observed proximity locations with the available proportions of each habitat use category within the mother–offspring home range with a Chi-square test with a significance level (alpha) set at 0.05.

## Results

### Distance between mother and fawn

**Fig. 3:**
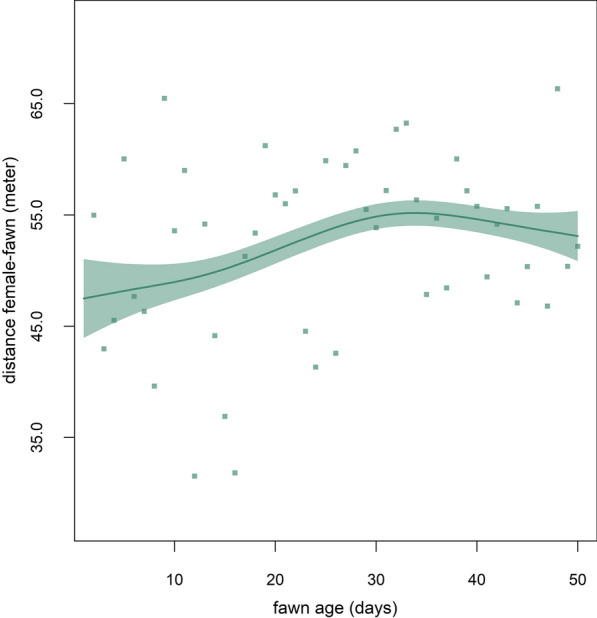
Predicted response curve describing the distance between a roe deer mother and her fawn during the first 50 days of fawn life based on a GAMM. Points represent the mean distances across all mother–offspring pairs (n = 13) per day. Y-axis values are back-transformed to meter

The age of the fawn influenced the observed distance between mother and offspring described by the smooth term of the GAMM. The median distance between a mother and her fawn over the first 50 days of life was 53.3 m, ranging between 35.9 and 162.2 m among individual pairs (Fig. 3). The distance increased for the first 36 days and decreased slightly thereafter.

### Timing of interactions between mother and fawn

**Fig. 4:**
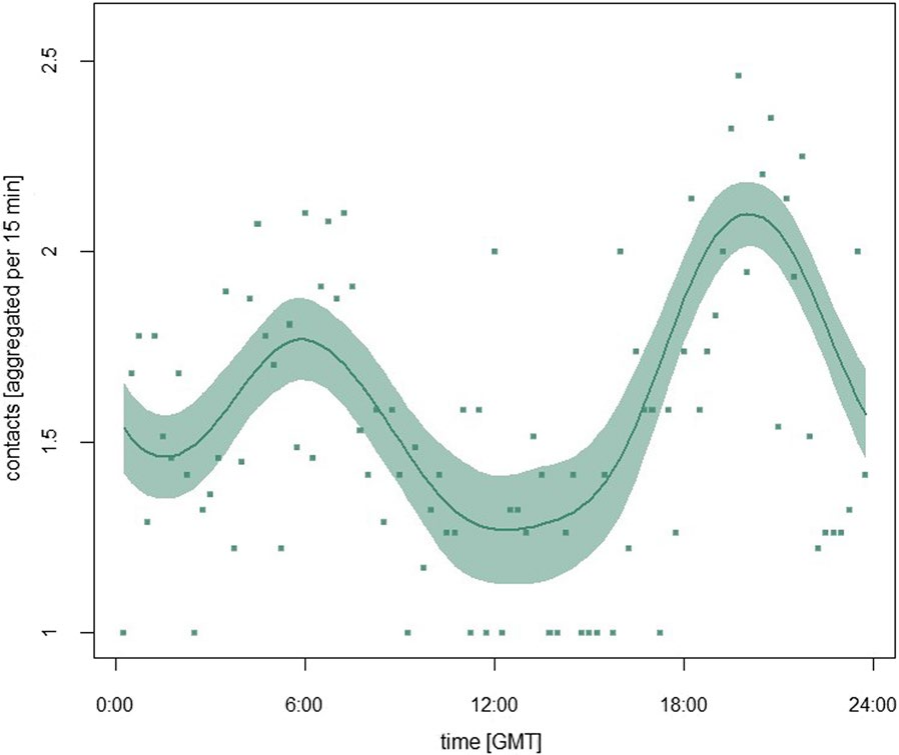
Predicted response curve describing the number of contacts per 15 min period between roe deer mother-fawn pairs over the 24 h cycle during the first 50 days of fawn life based on a GAMM with a cyclic spline of the time of the contact. The points represent the average number of contacts per 15 min across all roe deer mother-fawn pairs (n = 14)

We found strong variation in the number of proximity contacts between a roe deer mother and her fawn in relation to time of the day (Fig. 4). The highest number of contacts was recorded during dusk and dawn. The relative frequency of interactions was 4.8% higher during dawn, and 19.3% higher during dusk, compared to the daytime, accounting for the varying durations of each light phase.

### Relative habitat use of mother versus fawn

**Fig. 5:**
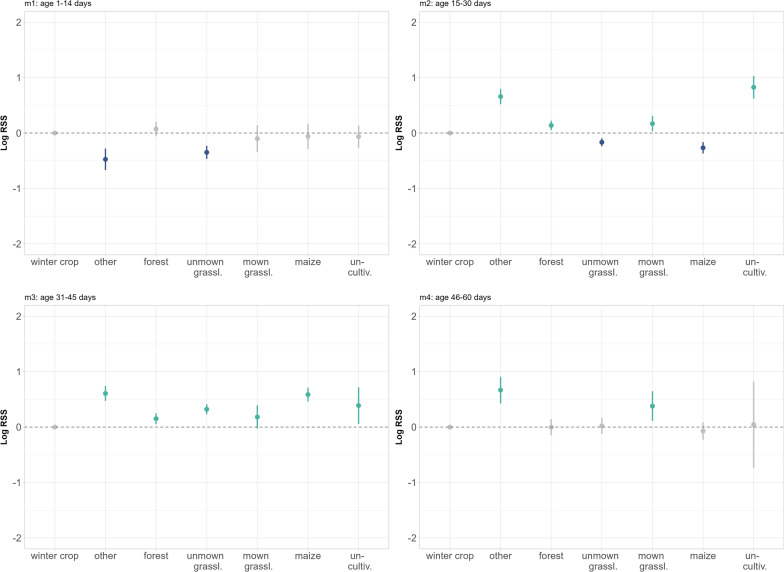
Relative selection strength (log-RSS) for habitat use of roe deer mothers relative to their fawns for each landcover type, while holding all other variables in the model constant and setting the reference category to *winter crops*. The colors indicate whether a variable was used significantly relatively more by mothers (green) or by fawns (blue). If the difference in use by mothers and fawns was not significant, it is colored gray

The latent selection difference functions suggested that the difference in relative habitat use between a mother and her fawn varied in relation to the fawn´s age-class (m1: age 1–14 days, m2: age 15–30, m3: age 31–45, m4: age 46–60; Additional file: Table A3, Fig. 5). *Unmown grassland* was used relatively more by fawns during the first month of life in relation to the reference category *winter crops* (m1: ß = -0.349; SE = 0.060; *p* = < 0.001; m2: ß = -0.167; SE = 0.037; *p* = < 0.001) compared to their mothers. Fawns of older age-classes used unmown grassland relatively less than mothers, although this difference was only significant during the m3 age-class (ß = 0.322; SE = 0.046; *p* = < 0.001). In contrast, *mown grassland* was relatively used more by mothers, although there were significant differences during two periods only (m2: ß = 0.170; SE = 0.070; *p* = 0.016; m4: ß = 0.379; SE = 0.136; *p* = 0.005). *Maize* was relatively less used by mothers than by fawns during m2 (ß = -0.267; SE = 0.053; *p* = < 0.001), whereas mothers used it relatively more in relation to the reference category during m3 (ß = 0.587; SE = 0.063; *p* = < 0.001). The relative use of forest did not differ significantly between mother and fawn during m1 or m4, but *forest* was used relatively more by mothers than fawns during the intervening four weeks (m2: ß = 0.138; SE = 0.043; *p* = 0.002; m3: ß = 0.152; SE = 0.050; *p* = 0.002). Fawns showed significantly higher relative use of the landcover type *other* during m1 (ß = − 0.475; SE = 0.100; *p* = < 0.001), while mothers used this landcover type relatively more compared to fawns the rest of the time (m2: ß = 0.658; SE = 0.071; *p* = < 0.001; m3: ß = 0.606; SE = 0.069; *p* = < 0.001; m4: ß = 0.668; SE = 0.123; *p* = < 0.001).

**Fig. 6:**
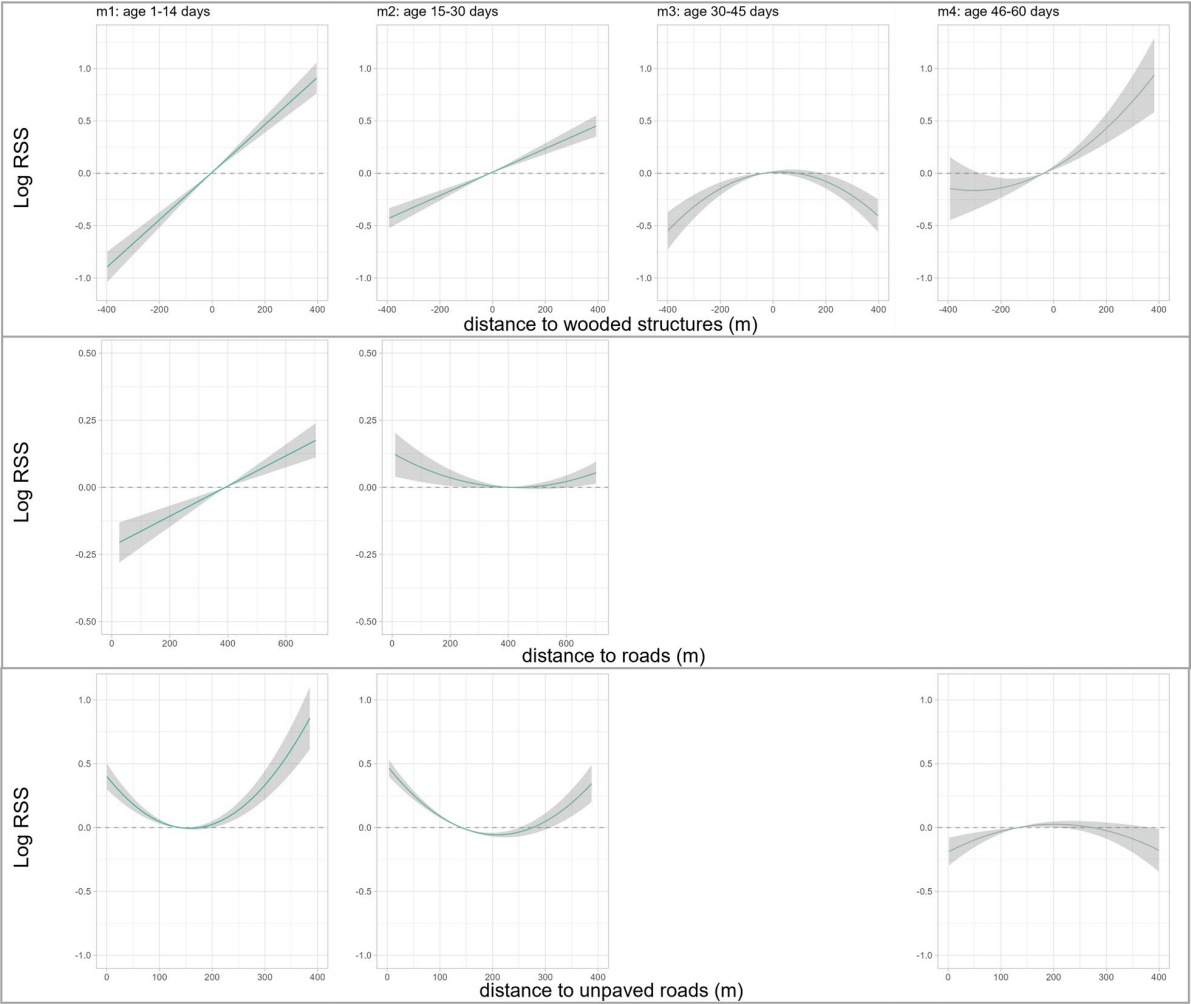
Relative selection strength (log-RSS) with 95% confidence intervals for significant explanatory variables. The graph shows the RSS for a location used relatively more by roe deer mothers across the range of possible distance values when fawn locations are held constant to their mean value (see Additional file: Table A2). Positive values indicate a higher use relative to the reference fawn location, negative values indicate avoidance

All models included positive ß-coefficients for the distance to roads, implying that mothers maintained a higher distance to roads relative to their fawns, although this effect was significant only during the first month of life (m1: ß = 0.1045; SE = 0.020; *p* = < 0.001; m2: ß = 0.029; SE = 0.008; *p* = < 0.001). Regarding the relative selection strength (RSS) for the distance to the road, mothers showed higher relative use of greater distances compared to the average distance of the fawns (370.2 m) during age classes m1 and m2. Additionally, during age class m2, mothers used short distances to roads relatively more frequently than the average fawn, but during m1, they avoided being closer to the road (Additional file: Table A3 and Fig. 6).

The LSDs suggested a similar influence regarding the distance to unpaved roads during the first month of the fawns’ life (m1 and m2). Considering the variable’s non-linearity, mothers used significantly greater distances from unpaved roads relative to their fawns. Additionally, they showed higher relative use of areas closer to unpaved roads compared to the average distance used by fawns of 142.9 m (m1: ß = 0.129; SE = 0.016; *p* = < 0.001) and 145.5 m; (m2: ß = 0.093; SE = 0.009; *p* = < 0.001). During m4, the situation was inversed, such that mothers avoided being closer to unpaved roads relative to the mean fawn location (Additional file: Table A3 and Fig. 6).

Lastly, the difference between mothers and fawns in terms of distance to wooded structures was significant across all age classes (Additional file: Table A3, m1: ß = 0.333; SE = 0.027; *p* = < 0.001; m2: ß = 0.165; SE = 0.019; *p* = < 0.001; m3: ß = -0.075; SE = 0.0100; *p* = < 0.001, m4: ß = 0.042; SE = 0.017; *p* = 0.012). Mothers maintained a significantly higher distance to wooded structures relative to their fawns´ mean location during m1 (2.33 m), m2 (− 15.5 m), and m4 (− 33.0 m) and, in comparison, avoided using locations that were deeper inside the forest patch. During m3, locations that were far from the forest edge in both directions were used relatively less by mothers compared to fawns (Fig. 6). The average distance to wooded structures, unpaved, and paved roads for mothers and fawns per age-class are summarized in the Additional file (Table A2).

### Habitat use at locations for mother–offspring interactions

**Fig. 7:**
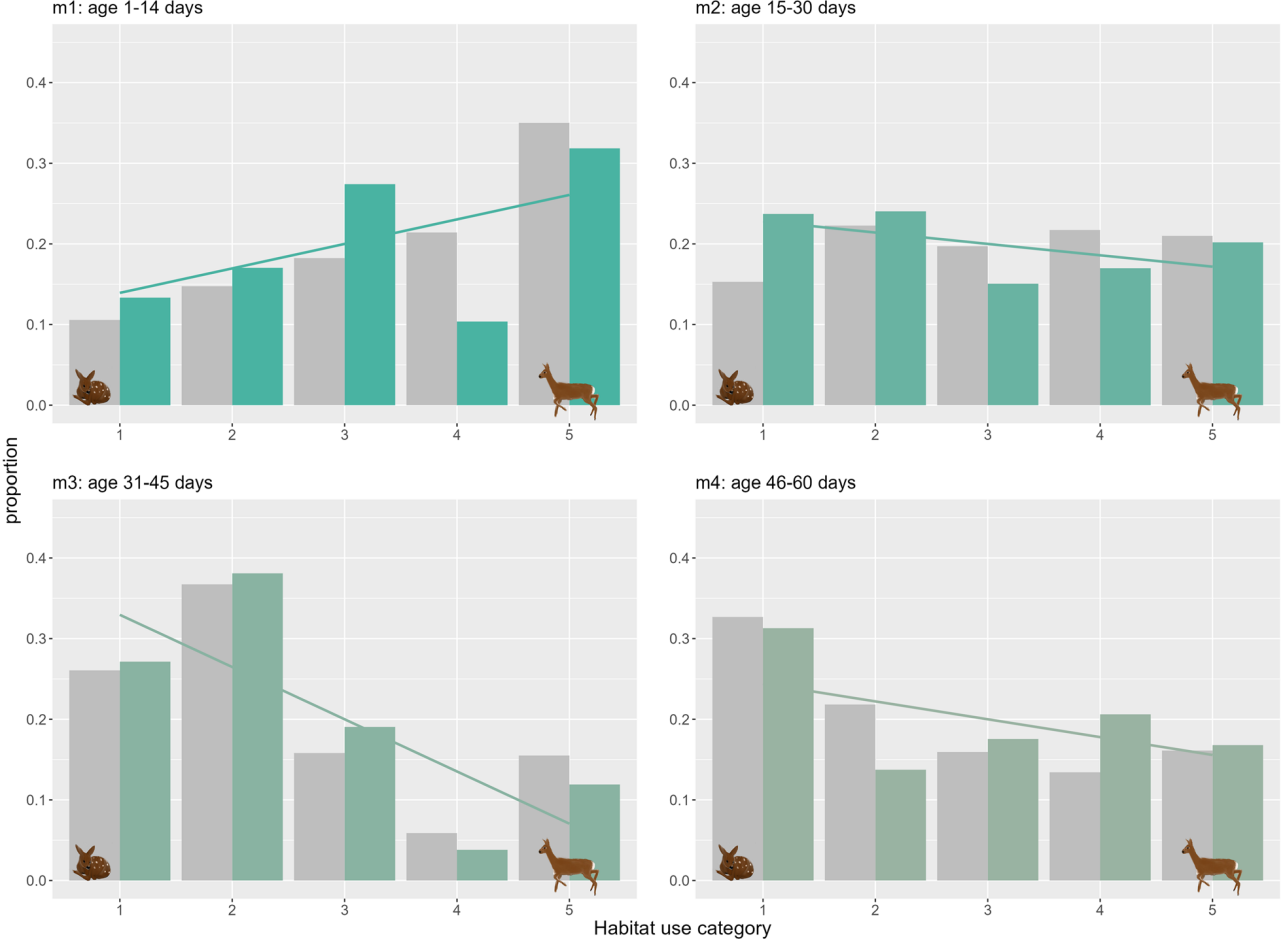
Proportion of the observed contacts between roe deer mother and her fawn per habitat use category (green) relative to their availability within each mother–offspring home range (gray). Habitat use categories closer to 1 primarily reflect the habitat preferences of fawns, and conversely, values closer to 5 indicate a high relative preference of mothers

We predicted the distribution of habitats for mother–offspring interactions by overlaying the spatially predicted LSD outputs with the proximity GPS locations (n = 789) (observed) and comparing them with expected values based on the relative availability of each habitat category within the home range of each mother–offspring pair (see Fig. 7). During m1, encounters happened more frequently in the intermediate habitat category (habitat that is equally used by mothers and fawns) and habitats used relatively more by mothers, but slightly more frequent in the categories used relatively more by fawns, given the availability of habitats. During m2, the locations for contacts were quite evenly distributed across the habitat categories, but again, we found a higher frequency of interactions in fawn-preferred habitats compared to their availability within the mother–offspring home range. In the second month of life (m3 and m4), the frequency of mother–offspring interactions shifted to habitats mainly favored by fawns. Note, however, that none of these differences among habitat categories was statistically significant (see Additional file: Table A4 for *p* values of the *χ*^2^-Test).

Regarding the habitat types where interactions occurred, mother and offspring met most often in forest habitat, and this was relatively invariable across the 60-day monitoring period. In contrast, interactions occurred decreasingly often in grassland over time, while they occurred increasingly often in winter crops. Interactions occurred only rarely in other crop types, although their occurrence in maize increased slightly over time until shortly before the end of the monitoring period (Additional file: Figure  A1).

## Discussion

For the first time, we used cutting-edge proximity loggers to track roe deer mothers and their dependent neonate hider offspring to record mother–offspring interactions over their first weeks of life. Using these high-resolution data, we provided empirical evidence that interactions, including bouts of maternal care, by roe deer follows the expected pattern for a hider species. Mothers and fawns spent most of their time separated, at a median distance of 53.3 m, which gradually increased as the fawn aged. Interactions between mother and offspring occurred more frequently during dusk and dawn, matching the habitual circadian rhythm of adult females. We found fine-scale differences in habitat use between mothers and fawns, but no significant differences when comparing the used habitats of does or fawns to those actually used during mother–offspring interactions.

### How far is a mother from her offspring?

Establishing a strong bond between the doe and her fawn is crucial for fawn survival [50, 54]. Consequently, for hider ungulates, the distance between the mother and her hidden fawn are short, especially during the first days of life [59]. In our analysis, mother-offspring distance was variable among pairs, but as expected, it significantly increased with increasing fawn age, before decreasing slightly after ∼30 days. This is in line with the results of Linnell et al. [59], who also found a very similar pattern, while Espmark [29] recorded a mean resting distance of ∼40 m during the first 50 days. This age-related change is likely associated with higher activity levels of fawns as they grow older and become increasingly independent [59]. Subsequently, this is followed by alignment in space use of the mother-offspring pair towards the end of our period of investigation (50 days), when the fawn progressively starts to synchronize activity with its mother, prior to the subsequent mating period in mid-summer.

Further, it has been shown that predators are less successful when newborns are defended by their mothers in ungulates [43, 56], although fawn survival also depends on the mother´s level of proactivity [63] and her experience [61, 70]. In general, the mortality rate of hiding ungulates is relatively high, especially during the first weeks of life and when offspring become more active [1, 19, 57]. For that reason, staying fairly close to the neonates´ bed site seems an appropriate anti-predator tactic for mothers of hider offspring. However, especially in open habitats, predators that use a sit-and-wait hunting tactic may be able to locate the fawns´ hiding place by observing the mother [43]. Hence, the mother’s ability to conceal information about her hiding neonates´ location is crucial for a successful hiding tactic [19]. In this regard, ungulate mothers often approach their fawns slowly, with elevated vigilance [12]. Recent work suggested that, the timing of maternal care can influence the survival of their neonates [67].

### When does a mother interact with her offspring?

With the aid of state-of-the-art proximity biologgers, we were able to report an unbiased estimate of the circadian rhythm of mother–offspring interactions. Indeed, previous studies have shown that, compared to human observers, proximity loggers detect contacts more accurately [88] and variation in detection parameters is low [75]. As expected, the timing of mother-fawn interactions matched the common bimodal daily activity patterns of roe deer females [9], as shown for other hider species [13]. Interestingly, interactions were observed more frequently in the evening than in the early morning, aligning with the activity patterns of reproductive does [9].

### Do mothers and fawns use different habitats?

Female ungulates are selected to prioritize self-maintenance over allocation to current reproduction, thus, maximizing longevity and, hence, the number of reproductive attempts [33]. However, the habitat choice of a mother with dependent young is crucial for her neonate´s development, as habitat quality and the mothers’s nutritional condition during early life influence birth weight, and body development and, thus, determine the offspring´s future reproductive potential [41, 52, 74]. Given that fawns almost exclusively use locations within to their mothers’ home range, we suggest that fawns were constrained to select their bed sites at a fine spatial scale within the preferred habitats of their mothers. Nevertheless, we identified some differences in relative habitat use between mothers and fawns. Specifically, in line with our prediction, fawns used open areas (unmown grassland) relatively more often than their mothers during the first four weeks of their life. These unmown fields offer high cover potential and are frequently used by roe deer neonates to hide, despite a potentially higher predation risk [72]. As fields in the surroundings are progressively mown, the abundance of fawns seeking cover within the remaining unmown fields increases [7]. Linnell et al. [58] showed that the use of agricultural crops by fawns tracks the phenological process of growth and harvest. However, given the high mortality rates of hiding fawns due to mowing activities under current agricultural land use practices, hiding behaviour in these crops may be considered an evolutionary trap [11, 34, 42]. As soon as fields have been mown, they no longer offer protective cover, however, mowing may increase plant growth and productivity [90], so that mothers increased their relative use of agricultural fields compared to their fawns, especially grassland. Lactating females need to spend more time foraging because of their high energetic requirements [22, 69] and forage quality is known to be high in agricultural habitats [39]. This is particularly the case for an income breeder (sensu [45]), such as the roe deer [5], that finances reproductive allocation through current intake. Indeed, the energetic costs of rearing young in roe deer, generally twins, are among the highest in a large wild herbivore [78]. Therefore, we suggest that roe deer mothers prioritize access to freshly regrown vegetation post-harvest to fulfill their very high energy requirements, despite the low cover for refuge [9].

We found that mothers used forest habitats relatively more compared to their fawns, presumably for cover. Indeed, in mixed agricultural landscapes, refuge habitats are crucial to avoid human disturbance during the day [14]. Concordantly, the hiding tactic for mothers and offspring seems to be the driving factor in their use of maize fields [77], in addition to the high availability in our northern study areas. Indeed, mothers used maize significantly less relative to fawns during the second half of their first month of life, but subsequently used it relatively more. For adult roe deer, the selection of crop types depends on both the food and cover potential in relation to the time of day and vegetation phenology [77]. Our observations corroborate this pattern, as maize is typically sown in our study areas from the beginning to mid-May. Therefore, maize provides sufficient cover to hide for offspring after the first few weeks post-partum and subsequently later for adult roe deer.

Because roe deer are known to avoid human infrastructure, which is perceived as a source of disturbance [23], compared to their mothers, we expected fawns to hide further from roads, especially unpaved ones, due to potential disturbance and risk associated with them. In particular, we expected the unpaved roads used by walkers with and without (unleashed) dogs to have a strong influence [62] also on bed site selection of fawns. In line with these expectations, fawns remained at intermediate distances from roads, whereas relative to their mothers, who often used areas both near to and far from unpaved roads, although interestingly, this pattern reversed towards the end of the monitoring period. As fawns are quite active after 6 weeks of age [59], this may reflect an age-dependent change in their habitat use, or a season-dependent change in the recreational use of trails by humans and agricultural activities during summer. Information on the frequency of use of roads by humans could shed more light on this question since the intensity of road use has an influence on the space use of large herbivores ([53], *Rangifer tarandus caribou*). In extreme cases, repeated disturbances may drive mothers to stop allocating to their neonates [81].

Roe deer are known to prefer habitat edges within their home range [85]. The differences between the used locations of roe deer mothers and fawns regarding the distances to the nearest wooded structure showed that mothers favored higher distances from the forest to the forest-field border, relative to fawns, who tended to be more often inside the forest. This effect was present in all models except between 31 and 45 days. Several studies have shown that habitat edges can have a positive influence on offspring survival rates and become more important with increasing age and activity of the neonates [49, 79, 86]. Thus, in addition to the concealment aspect for the fawns, habitat edges in fragmented landscapes offer mothers attractive foraging possibilities [39, 85].

### Where does a mother interact with her offspring?

Because female ungulates follow a conservative maternal care tactic, prioritizing their own survival and maintenance over allocation to their fawns [33], we expected, to observe most mother–offspring interactions in habitat use categories that are preferred by mothers, especially during the first weeks of fawns’ life. Our results only partly aligned with these initial predictions. We observed, as expected, a slight shift in habitats used for mother–offspring interactions from habitats preferred by mothers to habitats preferred by fawns, but we found no significant differences when relating this to the available habitat within the mother–offspring home ranges. We argue that, especially during the early stages of fawn life, mothers constrained the fawns to choose bed sites that primarily met their own habitat requirements. Thus, we speculate that roe deer mothers initially prioritize their own habitat requirements over those of their fawns [30, 84].

After 2–3 weeks post-partum, fawns actively seek interactions with their mothers and begin to move more independently between habitats [17]. This increased mobility and activity possibly lead to mother–offspring interactions occurring almost equally often across all habitats. The tendency for mother and offspring to meet more frequently in habitats preferred by fawns over their mothers increased in the second month of the fawns’ lives. Our approach has been used to contrast habitat use of different species (e.g. [26, 73]) for which space use generally differs more than between offspring and mother in a hider species.

Mothers interact with their offspring most often in forest habitat, which corroborates findings by Panzacchi et al. [72]. As the vegetation phenology progressed and fawns aged over the study, the cover potential provided by winter crops increased. As a result, mother–offspring interactions occurred more often in winter crop fields, while grassland was used less [58]. We suggest that this is due to a preference for interacting and providing care at locations with high concealment and, therefore, low detectability for predators. Accordingly, concealment has been identified as a key factor driving the selection of bed sites for both roe deer and ungulates in general [7, 36, 37, 47].

## Electronic supplementary material

Below is the link to the electronic supplementary material.

Supplementary Material 1

## Data Availability

The datasets analyzed in this study are currently not publicly available due to further ongoing analysis but are available from the corresponding author upon reasonable request.
