# Supplementary material for: The timing and spatial distribution of mother–offspring interactions in an obligate hider
Source: Mov Ecol. 2024 Nov 26;12:73. doi: 10.1186/s40462-024-00514-5 (PMC11590307; doi:10.1186/s40462-024-00514-5)
Supplement: Supplementary file 1 — Supplementary Material 1 [file 40462_2024_514_MOESM1_ESM.docx]

**Appendix:**


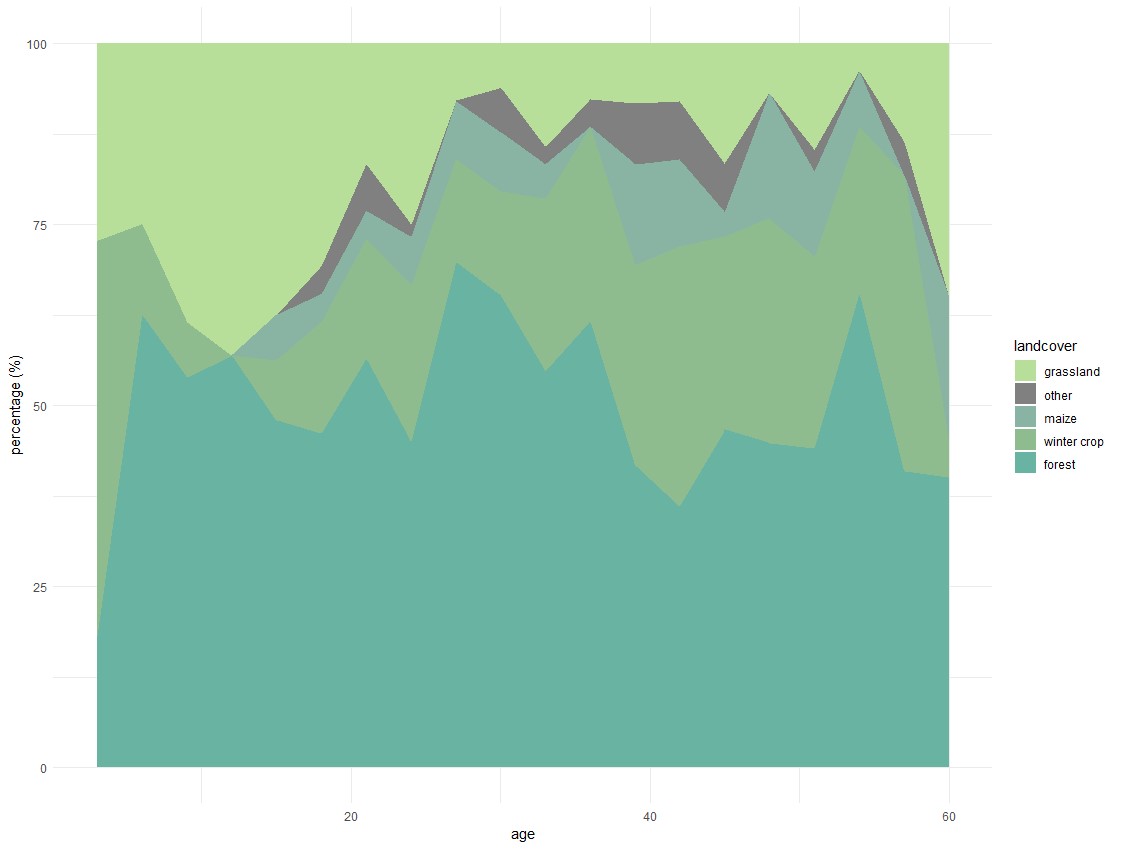


Figure A 1: Proportional use (per 3-day interval) of habitat types by roe deer females and their fawn at the locations of interactions (proximity locations) during the first 60 days of the fawn´s life.

Table A 1: Details of the roe deer female-fawn pairs monitored, in terms of location, year, length of monitoring, and analysis carried out.

|  |  |  |  |  |  |  | **analysis** | |  |
| --- | --- | --- | --- | --- | --- | --- | --- | --- | --- |
| **year** | **study area** | **pairID** | **age collaring (days)** | **data length (days)** | **data type** | **spatial** | **temporal** | **LSD** | **bins** |
| 2021 | Oettingen | i | 2 | 127 | GPS | X |  | X |  |
| 2021 | Oettingen | n | 17 | 90 | proximity, GPS | X | X | X | X |
| 2021 | Hagenau | q | 18 | 102 | proximity, GPS | X | X | X | X |
| 2021 | Oettingen | v | 10 | 78 | proximity, GPS | X | X | X | X |
| 2021 | Oettingen | h | 11 | 100 | GPS | X |  | X |  |
| 2021 | Hagenau | l | 4 | 103 | proximity, GPS | X | X | X | X |
| 2021 | Oettingen | t | 2 | 30 | proximity, GPS |  | X | X | X |
| 2021 | Oettingen | k | 7 | 39 | proximity, GPS |  | X | X | X |
| 2021 | Oettingen | p | 15 | 128 | proximity, GPS | X | X | X | X |
| 2021 | Hagenau | r | 9 | 152 | proximity, GPS | X | X | X | X |
| 2022 | Oettingen | f | 2 | 18 | proximity, GPS |  | X | X | X |
| 2022 | Hagenau | d | 2 | 77 | proximity, GPS | X | X | X | X |
| 2022 | Oettingen | b | 6 | 45 | proximity, GPS |  | X | X | X |
| 2022 | Steingaden | c | 4 | 158 | GPS | X |  | X |  |
| 2022 | Oettingen | a | 15 | 10 | GPS |  |  | X |  |
| 2022 | Oettingen | zz | 7 | 31 | proximity, GPS |  | X | X | X |
| 2022 | Hagenau | e | 4 | 33 | proximity, GPS |  | X | X | X |
| 2022 | Oettingen | j | 5 | 42 | GPS |  |  | X |  |
| 2023 | Oettingen | x | 1 | 118 | GPS | X |  | X |  |
| 2023 | Oettingen | z | 10 | 40 | GPS |  |  | X |  |
| 2023 | Oettingen | y | 14 | 138 | proximity, GPS | X | X | X | X |

Table A 2: Mean values of distance to wooded structures, roads, and unpaved roads of roe deer mothers and fawns per age class.

| **who** | **model**  **age:** | **distance to wooded structures (m)** | **distance to roads (m)** | **distance to unpaved roads (m)** |
| --- | --- | --- | --- | --- |
| fawn | 1-14 | 2.33 | 364.08 | 143.93 |
| female | 1-14 | -15.38 | 362.54 | 122.14 |
| fawn | 15-30 | -15.54 | 370.20 | 145.54 |
| female | 15-30 | 3.16 | 362.47 | 122.30 |
| fawn | 31-45 | -32.96 | 328.68 | 140.56 |
| female | 31-45 | 1.36 | 315.81 | 126.08 |
| fawn | 46-60 | -33.95 | 302.42 | 125.42 |
| female | 46-60 | -5.65 | 307.18 | 141.82 |

Table A 3: Latent selection difference (LSD) function contrasting relative habitat use of roe deer mothers (coded as 1) and fawns (coded as 0) during the first 60 days of fawn life (each model describes a 14-day period).

| **Model 1: age 1-14** | **Estimate** | **Std. Error** | **Z Value** | **P value** | **Sign. code** |
| --- | --- | --- | --- | --- | --- |
| (Intercept) | -0.013 | 0.057 | -0.223 | 0.823 |  |
| landcover: other | -0.475 | 0.100 | -4.774 | <0.001 | *** |
| landcover: forest | 0.072 | 0.067 | 1.073 | 0.283 |  |
| landcover: unmown grassland | -0.349 | 0.060 | -5.780 | <0.001 | *** |
| landcover: mown grassland | -0.102 | 0.125 | -0.814 | 0.416 |  |
| landcover: maize | -0.061 | 0.116 | -0.522 | 0.601 |  |
| landcover: uncultivated | -0.065 | 0.102 | -0.640 | 0.522 |  |
| distance to roads | 0.105 | 0.020 | 5.390 | <0.001 | *** |
| distance to unpaved roads | -0.090 | 0.019 | -4.733 | <0.001 | *** |
| I(distance to unpaved roads^2) | 0.129 | 0.016 | 8.181 | <0.001 | *** |
| distance to wooded structures  **Model 2: age 15-30** | 0.333 | 0.027 | 12.143 | <0.001 | *** |
| (Intercept) | -0.172 | 0.034 | -5.057 | <0.001 | *** |
| landcover: other | 0.658 | 0.071 | 9.304 | <0.001 | *** |
| landcover: forest | 0.138 | 0.043 | 3.185 | 0.002 | ** |
| landcover: unmown grassland | -0.167 | 0.037 | -4.491 | <0.001 | *** |
| landcover: mown grassland | 0.170 | 0.070 | 2.418 | 0.016 | * |
| landcover: maize | -0.267 | 0.053 | -5.027 | <0.001 | *** |
| landcover: uncultivated | 0.826 | 0.103 | 7.988 | <0.001 | *** |
| distance to roads | -0.017 | 0.013 | -1.393 | 0.164 |  |
| I(distance to roads^2) | 0.029 | 0.008 | 3.648 | <0.001 | *** |
| distance to unpaved roads | -0.176 | 0.013 | -13.961 | <0.001 | *** |
| I(distance to unpaved roads^2) | 0.093 | 0.009 | 10.083 | <0.001 | *** |
| distance to wooded structures | 0.165 | 0.019 | 8.832 | <0.001 | *** |

**Model 3: age 31-45**

| (Intercept) | -0.115 | 0.040 | -2.871 | 0.004 | ** |
| --- | --- | --- | --- | --- | --- |
| landcover: other | 0.606 | 0.069 | 8.840 | <0.001 | *** |
| landcover: forest | 0.152 | 0.050 | 3.037 | 0.002 | ** |
| landcover: unmown grassland | 0.322 | 0.046 | 7.027 | <0.001 | *** |
| landcover: mown grassland | 0.182 | 0.106 | 1.728 | 0.084 | . |
| landcover: maize | 0.587 | 0.063 | 9.286 | <0.001 | *** |
| landcover: uncultivated | 0.388 | 0.168 | 2.307 | 0.021 | * |
| distance to roads | 0.038 | 0.017 | 2.212 | 0.027 | * |
| I(distance to roads^2) | 0.020 | 0.017 | 1.173 | 0.241 |  |
| distance to unpaved roads | -0.015 | 0.015 | -0.982 | 0.326 |  |
| I(distance to unpaved roads^2) | -0.014 | 0.011 | -1.179 | 0.239 |  |
| distance to wooded structures | 0.033 | 0.023 | 1.447 | 0.148 |  |
| I(distance to wooded structures^2)  **Model 4: age 46-60** | -0.075 | 0.010 | -7.572 | <0.001 | *** |
| (Intercept) | 0.096 | 0.061 | 1.567 | 0.117 |  |
| landcover: other | 0.668 | 0.123 | 5.431 | <0.001 | *** |
| landcover: forest | -0.002 | 0.074 | -0.026 | 0.979 |  |
| landcover: unmown grassland | 0.022 | 0.073 | 0.303 | 0.762 |  |
| landcover: mown grassland | 0.379 | 0.136 | 2.781 | 0.005 | ** |
| landcover: maize | -0.071 | 0.080 | -0.893 | 0.372 |  |
| landcover: uncultivated | 0.045 | 0.397 | 0.114 | 0.909 |  |
| distance to roads | 0.030 | 0.024 | 1.290 | 0.197 |  |
| distance to unpaved roads | 0.068 | 0.022 | 3.083 | 0.002 | ** |
| I(distance to unpaved roads^2) | -0.052 | 0.017 | -3.127 | 0.002 | ** |
| distance to wooded structures | 0.182 | 0.032 | 5.791 | <0.001 | *** |
| I(distance to wooded structures^2) | 0.042 | 0.017 | 2.523 | 0.012 | * |
| Sign. codes: 0 '***' 0.001 '**' 0.01 '*' 0.05 '.' 0.1 ' ' 1 | | | | | |

Table A 4: P-values from the chi-square tests, which compare the relative proportions of habitat use categories at observed mother-offspring contact locations compared with the available proportions of each habitat use category within the mother-offspring home range during the first 60 days of the fawns’ lives (α = 0.05). For further details, see Figure 7 and the methods section.

| **age model** | **1-14** | **15-30** | **31-45** | **46-60** |
| --- | --- | --- | --- | --- |
| resource use category 1 | 0.9549 | 0.8927 | 0.9882 | 0.9862 |
| resource use category 2 | 0.9677 | 0.9793 | 0.9874 | 0.8921 |
| resource use category 3 | 0.8921 | 0.9372 | 0.9563 | 0.9777 |
| resource use category 4 | 0.8446 | 0.9393 | 0.9469 | 0.9020 |
| resource use category 5 | 0.9691 | 0.9900 | 0.9452 | 0.9906 |
